# Recent large-scale philophthalmosis outbreak in Portugal: inefficacy of common antihelminthic agents

**DOI:** 10.1186/s13071-022-05265-z

**Published:** 2022-05-12

**Authors:** Petr Heneberg, María Casero

**Affiliations:** 1 Third Faculty of Medicine, Charles University, Prague, Czech Republic; 2 Wildlife Rehabilitation and Research Center of Ria Formosa (RIAS), Ria Formosa Natural Park, Olhão, Portugal

**Keywords:** Avermectin, Conjunctivitis, Digenea, Freshwater snail, Helminth, Imidazothiazole, Macrocyclic lactone, *Philophthalmus lucipetus*, Population dynamics, Trematoda

## Abstract

**Background:**

Parasitic conjunctivitis caused by *Philophthalmus* spp. is a common ophthalmic disease in birds, with localized outbreaks occurring worldwide. There is no consensus on treating this disease; mechanical removal is considered a standard recommendation, but is associated with disease relapses within days or weeks.

**Methods:**

From 2015 to 2020, we examined 4295 *Larus michahellis* and *Larus fuscus* gulls in southern Portugal for the presence of *Philophthalmus* spp. Due to the need to treat dozens of infected gulls in the rescue station, we tested three treatment regimens aimed at targeting *Philophthalmus lucipetus* in the infected gulls: (I) the ophthalmic application of levamisole; (II) the oral application of milbemycin in combination with praziquantel; and (III) the subcutaneous application of ivermectin.

**Results:**

The outbreak of philophthalmosis in gulls in southern Portugal has been ongoing since the first cases were reported in 2015–2016. The prevalence of philophthalmosis has fluctuated annually, peaking a maximum of 10.3% in *L. fuscus* in 2017 and at 2.1% in *L. michahellis* in 2016. The infection intensity peaked at a median of 11.5 eye-flukes per host bird in *L. fuscus* in 2016 and a median of six eye-flukes per host bird in *L. michahellis* in 2017. Nine gulls were infected with >50 eye-flukes. None of the treatment options were effective at treating *P. lucipetus* infections: the numbers of eye-flukes in the infected birds did not decrease, and the clinical signs of the disease did not change.

**Conclusions:**

An outbreak of philophthalmosis in southern Portugal has massively affected two species of gulls in the region. Two previously suggested philophthalmosis treatments (ocular levamisole and praziquantel given orally), as well as a third mode of treatment with a previously failed compound (ivermectin administered subcutaneously) were used. However, the treatments did not affect the numbers of *P. lucipetus* in the eyes of the treated gulls. Further research should address ophthalmic gel formulations or sub-conjunctival delivery mode for antihelminthic drugs that are effective against *Philophthalmus* spp. in vitro.

**Graphical abstract:**

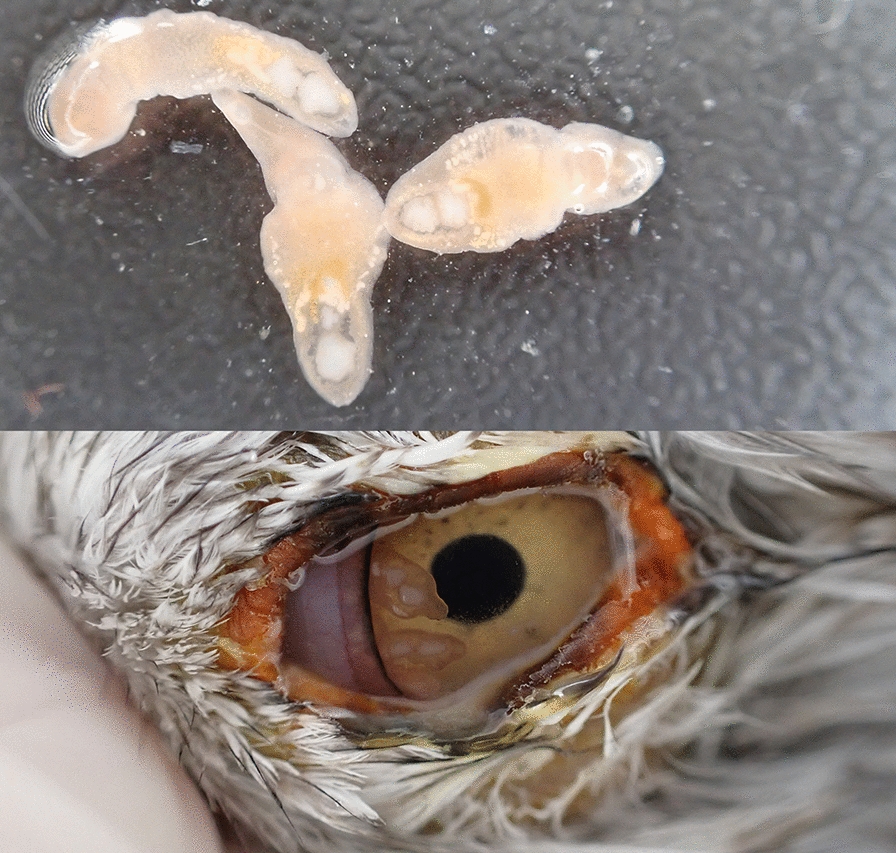

## Background

*Philophthalmus* spp. has been previously reported in multiple avian and mammalian species worldwide, including humans [1]. The intermediate hosts are freshwater snails, including the invasive *Melanoides tuberculata* [2]. Local outbreaks of *Philophthalmus* spp. can reach a prevalence of up to several dozen percent [3–6]. An outbreak of *Philophthalmus lucipetus* and *Philophthalmus lacrymosus* was reported in the lesser black-backed gull *Larus fuscus* and the yellow-legged gull *Larus michahellis* in southern Portugal since 2015 and included several dozen gulls [7]. While *P. lucipetus* did not cause any eye damage to the infected gulls examined, *P. lacrymosus* was found to have induced deep corneal ulcers and likely caused the absence of an eye in one of the examined gulls.

Attempts to treat *P. lacrymosus* infection have included the intramuscular (*i.m*.) injection and ophthalmic delivery of ivermectin in two doses over a 7-day period. However, the authors reported that this treatment had no effects [7]. The authors of another study reported that *i.m.* application of closely related doramectin (in combination with chloramphenicol as an ointment) to treat ostriches (*Struthio camelus*) infected by *Philophthalmus gralli* failed to produce the desired antiparasitic effects [8]. However, moxidectin, an ivermectin/clorsulon mixture, has been reported to be effective against other trematodes, such as *Fasciola* and *Paramphistomum* [9–12]. Contrasting experimental data were reported by Shoop et al. [13]. The ivermectin/clorsulon and ivermectin/closantel mixtures were partially effective against *Fasciola hepatica* in cattle in Mexico (effectiveness: 79–90% and 46–69%, respectively) [14]. The ivermectin/clorsulon/triclabendazole mixture had 90.9% effectivity against 4-week-old *F. hepatica* in cattle, while the effectivity of the ivermectin/clorsulon and ivermectin/closantel mixtures was much lower, at 29.7% and 26.8%, respectively. However, the effectivity was 99.9, 99.3 and 99.2% for all the three treatment modes, respectively, when tested against 12-week-old *F. hepatica* [15].

Several research groups have tested praziquantel as a treatment for philophthalmosis. Heneberg et al. [7] tested pyrantel, fenbendazole and praziquantel multitherapy for 3 days, supplemented by the daily mechanical removal of newly appearing eye-flukes. The authors reported that this combined pharmacotherapeutic and surgical treatment was insufficient at completely eliminating the eye-flukes [7]. Other *Philophthalmus* spp. have also been reported to cause ocular swelling, conjunctivitis, persistent lacrimation and ocular purulent discharge [16, 17]. Praziquantel effectively kills *P. gralli *in vitro when administered at 10 μg ml^-1^ for 24 h [18]. This anti-worm medication has also been used in vivo to treat captive rheas [19]. In this study, praziquantel was ocularly administered as a 1% ointment at 12-h intervals to rheas that were first subject to surgical removal of the eye-flukes. When Church et al. decreased the treatment frequency to a 24 h-cycle, 3 weeks later, two or three visible eye-flukes were on the dorsal conjunctival fornices. These authors continued the treatment for 20 consecutive weeks [19], which is a time frame that is unrealistic for wildlife rescue station settings. Moreover, 3 weeks after discontinuing the treatment, four to six eye flukes emerged again within the dorsal palpebral conjunctival fornix of the examined rhea. After an additional 2 weeks of ocular treatment with 1% praziquantel ointment at 12-h intervals, the rhea was finally free of the infection. Additional two rheas were treated using the same approach. In one of them, the surgical treatment combined with ocular treatment with 1% praziquantel ointment, at 12-h intervals, for 15 weeks resulted in a complete response. In the second case, the same treatment was ineffective, and the eye-flukes re-emerged in the dorsolateral conjunctival fornix and dorsomedial conjunctival fornix at weeks 3, 7 and 10. At week 15, this case was free of eye-flukes despite the treatment not including additional surgical removals beyond day 0 [19]. Assis et al. tested praziquantel against *P. gralli* [20]. In their study, they used four chickens experimentally infected with *P. gralli* and applied three sequential treatments with *i.m.* praziquantel followed by peroral fenbendazole. The praziquantel was administered at 10 mg kg^-1^ (post-infection day 120), 50 mg kg^-1^ (post-infection day 134) and 100 mg kg^-1^ (post-infection day 148), followed by peroral fenbendazole at 50 mg kg^-1^ (post-infection day 162) in three doses every 24 h. The treatment did not eliminate the eye-flukes [20].

Other treatment options tested to date include *i.m*. closantel (in combination with chloramphenicol as an ointment) in ostriches *S. camelus* infected by *P. gralli*, but this treatment was found to be ineffective [8]. Ophthalmically delivered levamisole (in combination with chloramphenicol as an ointment) led to a reduction in the infection burden in ostriches *S. camelus* infected by *P. gralli* by > 95% a week after the second levamisole treatment [8]. In total, four ostriches were treated: one displayed a complete response, two displayed a partial response and the fourth died due to an unknown cause [8]. Manual removal of *P. gralli* from the eyes of anesthetized ostriches was ineffective [21]. Finally, this species was also treated with carbamate powder in combination with an antibiotic, three times a day, which led to a temporary reduction in the number of eye-flukes, but new worms invaded the eyes within a few days. Additional carbamate treatment resulted in the elimination of the eye-flukes [21].

Here, we report philophthalmosis as a continuing health issue in gulls in southern Portugal. At the rescue station in Ria Formosa Natural Park (RIAS), we have received dozens of infected gulls, prompting the need for effective treatments. Because the simple surgical removal of eye-flukes is ineffective at preventing the regrowth of new eye-flukes, we tested three treatment regimens, namely: (I) the ophthalmic application of levamisole; (II) the oral application of milbemycin in combination with praziquantel; and (III) the subcutaneous application of ivermectin.

## Methods

From September 2015 to December 2020, we examined 4295 gulls for the presence of *Philophthalmus* spp., consisting of 2906 individuals of *L. michahellis* and 1389 individuals of *L. fuscus* that had been admitted for treatment for injuries or diseases at the Wildlife Rehabilitation and Research Center of Ria Formosa-RIAS (Olhão, Faro district) (Table 1). The authors and the hospital staff inspected the eyes of all birds at admission and release for the presence of *Philophthalmus* spp. We surgically removed these worms and counted them, except for birds that were later treated as specified below. The only exceptions to surgical removal of the worms were birds with paretic syndrome and those with eyelid paralysis, as both conditions are aggravating factors that make it difficult to control the worms in the eyes of the affected hosts. Consequently, the overall prevalence of *Philophthalmus* spp. in this study might be slightly underestimated. All of the examined birds originated from southern Portugal and up to October 2016 had already been included in a previous study that focused on the onset of the philophthalmosis outbreak and the identification of its causative agents to species [7].

In 2020, inspired by the randomized controlled trial by Mukaratirwa et al. [8], we randomly selected four gulls infected with *P. lucipetus* to be treated with ophthalmically delivered levamisole (study arm 1). In each treated bird, we recorded the number of visible *P. lucipetus* individuals at the start of the treatment but did not conduct surgical removal of the worms. As the worm bodies may overlap with one another in cases of high infection intensity, only approximate numbers of worms were recorded in the presence of high infection intensities. We used a commercial levamisole formulation (Tabernil Vermicida; Divasa-Farmavic, S.A., Barcelona, Spain) at 75 mg ml^-1^, and applied one drop (approx. 10 μl) of the compound to each eye every 7 days for either 4 (1 individual) or 5 (3 individuals) weeks. The hosts were monitored for a period of 5 (1 individual) or 6 (3 individuals) weeks.

The results of the levamisole treatment were unsatisfactory, and therefore we initiated the second arm of this randomized controlled study, which consisted of treating three randomly selected *P. lucipetus*-infected gulls with an orally administered mixture of milbemycin and praziquantel. We used a commercial praziquantel and milbemycin formulation (Milbemax; Elanco France SAS, Huningue, France) and administered Milbemax by oral catheter as half a tablet (final dose 12.5 mg of praziquantel and 1.25 mg of milbemycin per host) twice, on day 0 and day 14, and the monitored the hosts until day 21.

As both of the above-mentioned treatments had unsatisfactory outcomes, we initiated the third arm of this randomized controlled study by treating seven randomly selected *P. lucipetus*-infected gulls with ivermectin delivered subcutaneously (*s.c.*). A commercial formulation of ivermectin was used (Vectimax; Divasa-Farmavic, S.A., Barcelona, Spain) at 10 mg ml^-1^. We ivermectin was administered *s.c.* at a dose of 200 μg kg^-1^ twice, on day 0 and day 14, and the hosts were monitored until day 21.

## Results

### Outbreak of philophthalmosis

**Fig. 1:**
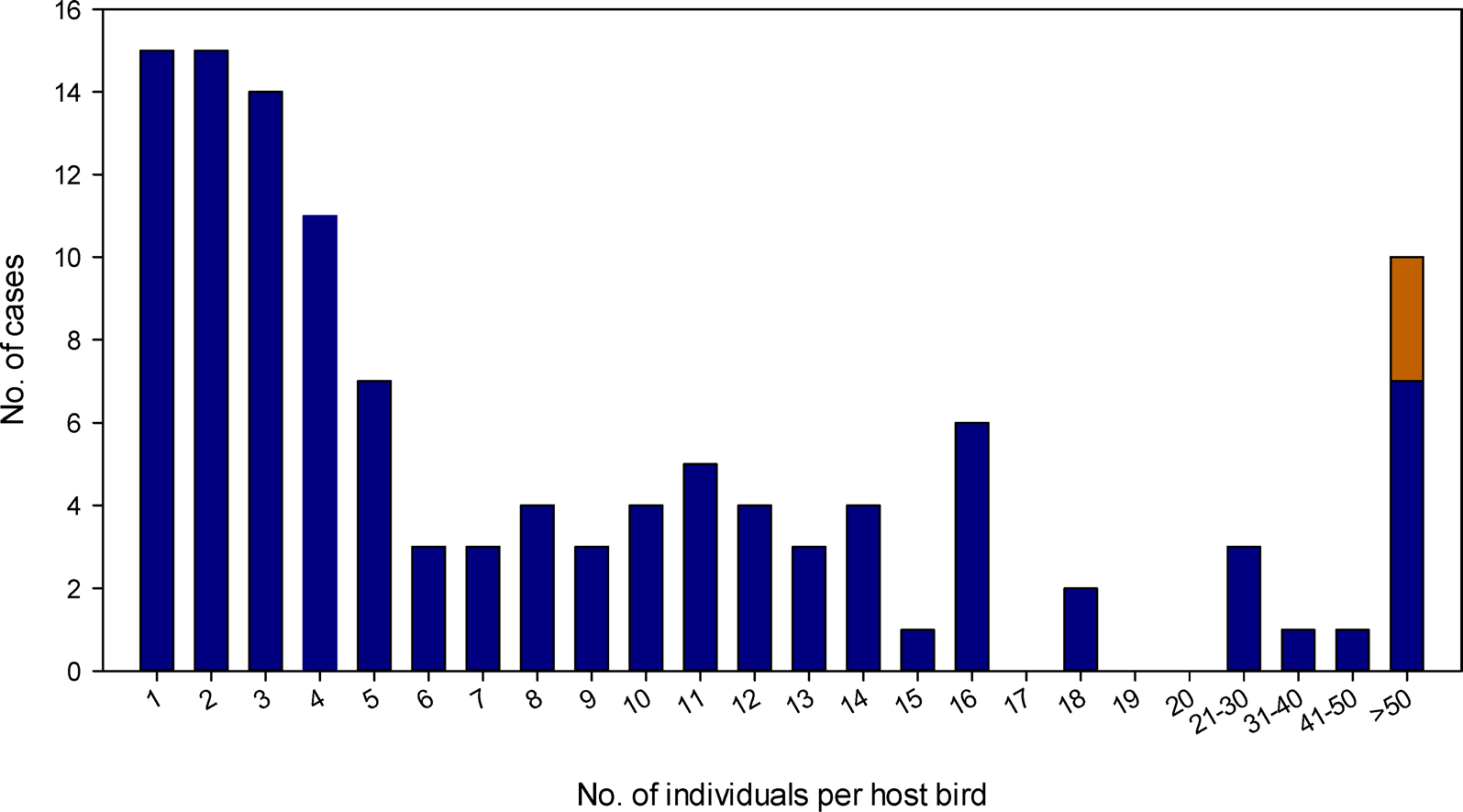
Intensity of infection by eye-flukes in the analyzed gulls (*Larus fuscus* and *Larus michahellis*). Infections by *Philophthalmus lucipetus* are shown in blue. The rare infections by *Philophthalmus lacrymosus* are shown in red

The outbreak of philophthalmosis in gulls has been ongoing since the first cases were reported in 2015–2016. The prevalence and intensity rates have varied over the years and differed between the two affected gull species (Table 1). The infection prevalence was higher in *L. fuscus* than in *L. michahellis*. In *L. fuscus*, the prevalence reached 5.7%, with a total of 76 infected birds found between 2015 and 2020. In *L. michahellis*, the prevalence reached 1.4%, with a total of 40 infected birds found between 2015 and 2020. The recorded prevalence in *L. michahellis* differed significantly from the expected *L. fuscus*-based prevalence of philophthalmosis (*χ*^2^ test, *P* < 0.001). In both bird species, the prevalence varied over time. In *L. fuscus*, the prevalence increased from 6.3% in 2015 to a maximum of 10.3% in 2017 and then decreased to 3.4% in 2019 and 4.0% in 2020. In *L. michahellis*, only one infected bird was found in 2015 and 2018, respectively, but the prevalence reached 2.1% in 2016 and 1.8% in 2020 (Table 1). Similarly, infection intensities were higher in *L. fuscus* than in *L. michahellis* (median infection intensity: 6 vs 3 eye-flukes per host bird). In both host species, the infection intensities were highly variable. Although the majority of the hosts were infected with only one or a few eye-flukes, we found over 50 eye-flukes per host in a total of nine gulls (Fig. 1). In all but three of these cases, the infection agent was *P. lucipetus*; two hosts in 2015 and one in 2020 were infected by *P. lacrymosus*.

**Table 1:** The numbers of host birds examined and infected by *Philophthalmus* spp. during the study period and intensity of infection

Year	Host species					
	*Larus michahellis*			*Larus fuscus*		
	Total* n* examined	Infected birds,* n* (%)	Intensity of infection^a^	Total* n* examined	Infected birds,* n* (%)	Intensity of infection^a^
2015	205	1 (0.5%)	2	126	8 (6.3%)	3.5 (1– >50)
2016	341	7 (2.1%)	2 (1–16)	199	18 (9.0%)	11.5 (1–76)
2017	396	6 (1.5%)	6 (1–50)	185	19 (10.3%)	6 (1- >50)
2018	339	1 (0.3%)	5	160	7 (4.4%)	4 (3–18)
2019	802	10 (1.2%)	3 (1–51)	322	11 (3.4%)	3 (2–15)
2020	823	15 (1.8%)	4 (1– >50)	397	16 (4.0%)	4 (1– >50)

^a^Median number of eye-flukes per host bird, with the range given in parentheses

### Philophthalmosis treatment

Three treatment regimens for *P. lucipetus* infection were tested: (I) the ophthalmic application of levamisole (study arm 1); (ii) the oral application of milbemycin in combination with praziquantel (study arm 2); and (3) the *s.c. *application of ivermectin (study arm 3). None of the treatments were effective, and during routine weekly inspections, we observed the same numbers of eye-flukes as at the start of treatment (Table 2). The treated birds also did not display any improvement in clinical signs of the disease. One bird in study arm 2 died 3 days after the final dose from unknown causes.

**Table 2:** Eye-fluke burden at day 0 (prior to the treatment), drugs administered for each case and treatment outcomes

Gull ID	Species	No. of worms at day 0		Treatment	Treatment outcomes				
		Right eye	Left eye		Day 7	Day 14	Day 21	Day 28	Day 35
*Study arm 1*									
1	*L. fuscus*	0	2	Levamisol	No change	No change	No change	No change	No change
2	*L. fuscus*	> 50	> 50	Levamisol	No change	No change	No change	No change	No change
3	*L. michahellis*	> 50	> 50	Levamisol	No change	No change	No change	No change	
4	*L. michahellis*	> 10	> 5	Levamisol	No change	No change	No change	No change	
*Study arm 2*									
5	*L. fuscus*	> 5	> 5	Milbemycin + praziquantel	No change	No change	Dead		
6	*L. fuscus*	0	1	Milbemycin + praziquantel	No change	No change	No change		
7	*L. fuscus*	> 5	> 5	Milbemycin + praziquantel	No change	No change	No change		
*Study arm 3*									
8	*L. fuscus*	> 5	> 5	Ivermectin	No change	No change	No change		
9	*L. michahellis*	0	1	Ivermectin	No change	No change	No change		
10	*L. fuscus*	< 5	< 5	Ivermectin	No change	No change	No change		
11	*L. fuscus*	5	5	Ivermectin	No change	No change	No change		
12	*L. fuscus*	10	10	Ivermectin	No change	No change	No change		
13	*L. fuscus*	< 5	< 5	Ivermectin	No change	No change	No change		
14	*L. fuscus*	1	2	Ivermectin	No change	No change	No change		

## Discussion

Parasitic conjunctivitis is difficult to treat. Common antihelminthics display inefficacy in the treatment of both *Philophthalmus*-induced conjunctivitis in birds and other types of parasitic conjunctivitis in ruminants [22–25]. Mechanical removal is considered to be a standard recommendation, but this treatment has been associated with disease relapses within days or weeks. Moreover, when treating animals in rescue stations, surgical removal is often associated with excessive stress. It is debatable whether the reduction in the parasitic load and parasite transmission to the environment outweigh the associated stress to the host. Previous research on ruminants led to similar conclusions as our experiments with infected birds [22]. Trials have shown that systemic antihelmintic drugs are mostly ineffective [23], while some local application modes lead to better treatment outcomes. The latter include the dermal application of doramectin [24] or the dermal application of a 10% imidacloprid and 2.5% moxidectin combination, which is an ivermectin/clorsulon mixture (imidacloprid alone was ineffective and moxidectin alone was not tested) [25]. However, the causative agent of bovine parasitic conjunctivitis is a nematode (Spirurida), whereas the causative agent of bird parasitic conjunctivitis is a trematode (Plagiorchiida); therefore, the treatment outcomes may differ.

In the present study, we were unable to confirm the promising data reported by Mukaratirwa et al. [8] who tested three types of philophthalmosis treatment and concluded that ophthalmically delivered levamisole is the best treatment option for the closely related *P. gralli*. To the contrary, in the present study we did not observe any response when treating four birds with philophthalmosis with repeated ophthalmically administered levamisole (Table 2).

Similarly, we were unable to confirm the promising in vitro data reported for praziquantel in the treatment of the closely related *P. gralli* [18]. In our in vivo trial, we did not observe any effect of praziquantel when administered in combination with milbemycin (Table 2). Praziquantel is toxic to *Philophthalmus *in vitro [18]. However, in a previous study it was found to be ineffective when used for 3 consecutive days in multitherapy with pyrantel and fenbendazole in vivo [7]. In addition, the previously reported in vivo administration of praziquantel alone against the closely related *P. gralli* in three captive rheas led to unsatisfactory results and worked only after very long treatment periods [19]. Milbemycins, which are agonists of glutamate-gated chloride channels, have been shown to act against nematodes and insects. However, a combined in vivo and in vitro study of milbemycins revealed a lack of activity against *F. hepatica* [13]. We did not investigate praziquantel as sole treatment, but it is unlikely that praziquantel monotherapy would produce more promising results. It is possible that the local topical administration of praziquantel might lead to a response when applied for several consecutive months, as reported previously [19].

In the third arm of the present study, we tested ivermectin delivered *s.c.* The authors of a previous study administered ivermectin *i.m.* and ophthalmically for the treatment of *P. lacrymosus* and did not observe any effect [7]. A derivative of ivermectin, doramectin, was also found to be ineffective when applied *i.m*. In the present study, we complement the above information with the *s.c. *administration of ivermectin, which also lacked any response.

### Limitations

It should be noted that the use of the oral formulation of levamisole (Tabernil Vermicida) should be considered to be only tentative. We followed the protocol suggested by Mukaratirwa et al. [8] as we attempted to corroborate the results reported in their paper. We closely monitored the treated birds for any signs of discomfort and did not find any. However, the eye has specific pH and osmolarity, and the use of oral formulations of drugs could, in general, be associated with discomfort and eye irritation. It should also be noted that the ocular bioavailability is very low with topical drop administration due to tear turnover, nasolacrymal drainage, reflex blinking and ocular static and dynamic barriers [26]. It is challenging to ensure drug delivery into posterior segment ocular tissues following topical eye drops instillation because of the above-mentioned barriers. The solutions to overcoming these bioavailability issues may emerge with the development of advanced ophthalmic gel formulations; also sub-conjunctival application could be tested when formulations with extended delivery are available [27].

Another limitation of this study are the uncertainties with the dosage of the compounds used. Regarding levamisole, the dosage used represented 0.75 mg of the compound. The recommended dosage for oral delivery of Tabernil Vermicida is 5.7 mg/day (when provided to an adult *L. fuscus* weighing approx. 770 g). Mukaratirwa et al. used a topical formulation of levamisole at 0.75 mg/kg to each eye and reported effective removal of eye-flukes after two applications [8]. Data for levamisole absorption are not available, but some compounds, such as the cholinergic parasympathomimetic agonist pilocarpine, are known to be absorbed across the conjunctiva and into the bloodstream at the striking rate of approximately 50% [28]. In chicken, intravenous (*i.v*.) administration of levamisole was tested and the removal of > 88% of *Capillaria* worms was reported when 36 mg/kg and 48 mg/kg levamisole was applied through the *i.v*. route [29, 30]. However, in birds, levamisole is quickly eliminated following *i.v.* application; the plasma concentration curve follows a three-compartment open model and the half-life is approximately 5.7 h [31]. The application of higher levamisole dose or the formulation of prolonged-acting levamisole could overcome this treatment inefficacy.

Regarding the ivermectin treatment, the formulation tested in the present study consisted of monotherapy alone. Data from *Philophthalmus*-infected individuals are scarce, but the data from *Fasciola*-infected cattle clearly show the benefit of multitherapy, in which ivermectin is used in combination with closantel or clorsulon and with triclabendazole [9–12, 14, 15]. These multitherapeutic treatment modes remain to be tested against *Philophthalmus* spp.

## Conclusions

An outbreak of philophthalmosis in southern Portugal has massively affected the gulls of two species in this region. Despite the widespread infections, there is no agreement on how to treat severely infected birds. Surgical removal of flukes combined with treatment with anti-inflammatory corticosteroids and preventive antibiotics is considered to be the first-line treatment. However, disease relapses are common in highly infected hosts. Here, we tested two previously suggested treatments of philophthalmosis (ocular levamisole and praziquantel administered orally) as well as another mode of treatment with a previously failed compound (*s.c*. ivermectin). However, the treatments did not affect the numbers of worms in the eyes of the treated gulls. Further research should address the delivery mechanisms of known antihelmintics to the eye and ocular parasites. The eye is a privileged organ, and blood–ocular barriers complicate systemic and topical treatments of ocular disorders [32]. Advanced ophthalmic gel formulations or subconjunctival application of formulations with extended drug delivery have yet to be tested in the treatment of philophthalmosis with antihelminthic drugs that are effective against eye-flukes in vitro.

## Data Availability

All data are available in the main text.
